# Of the few Black coaches in Brazilian professional basketball leagues: approaches to racism

**DOI:** 10.3389/fpsyg.2025.1511967

**Published:** 2025-04-10

**Authors:** Julia Oliveira Marcelino, Bartira Pereira Palma, Larissa Rafaela Galatti

**Affiliations:** ^1^Laboratório de Estudos em Pedagogia do Esporte – LEPE, Faculdade de Ciências Aplicadas, Universidade Estadual de Campinas, Limeira, Brazil; ^2^Faculdade de Educação Física, Universidade Estadual de Campinas, Campinas, Brazil

**Keywords:** sports, prejudice, professional career, coach development, social justice

## Abstract

**Introduction:**

Within Brazilian sports, Black individuals are significantly underrepresented in leadership roles, a disparity especially evident in elite sporting spheres, which remain highly exclusive. This study aimed to investigate the perceptions of Black elite basketball coaches regarding the impact of racism on their professional trajectories within Brazilian professional basketball leagues.

**Methods:**

Semi-structured interviews were conducted with four Black professional basketball coaches (two men and two women) working or who had worked in Brazil’s primary leagues, the NBB (Novo Basquete Brasil—New Basketball Brazil) and the LBF (Liga de Basquete Feminino—Women’s Basketball League). Data were analyzed using reflexive thematic analysis.

**Results:**

Five broad themes and seven subthemes emerged from the narratives: (1) Support Network, encompassing the individuals, events, and environments that shaped their careers; (2) Combating Racism, detailing strategies developed to cope with racism, with two subthemes: (a) Blaming the Victim, and (b) Dealing with Racism; (3) Barriers, describing obstacles that complicated their professional journeys, with three subthemes: (a) Questioning Professional Competence, (b) Disadvantages, and (c) Perception of Structural Racism; (4) Black Individuals in Leadership Roles, emphasizing the importance of visibility and representation, with two subthemes: (a) Identity, capturing their self-recognition as Black individuals, and (b) Importance of Black Individuals in Leadership Roles; and (5) Accumulation of Prejudices, highlighting the compounded effects of racial and other intersecting biases.

**Discussion:**

The findings emphasize the urgent need for policies promoting a more inclusive and equitable environment for Black basketball coaches in Brazil. Tailored interventions addressing the unique challenges encountered by Black male and female coaches could mitigate the impacts of structural racism. Implementing such strategies could foster a more just landscape within Brazilian basketball, providing Black coaches with enhanced opportunities to succeed and contribute in leadership roles.

## 1 Introduction

In Brazil, over 55% of the population identifies as light-skinned Black or Black (i.e., Black individuals); however, more than 68% of leadership positions are held by individuals who identify as White (Brazilian Institute of Geography and Statistics—IBGE, 2018, 2023). This scenario can be explained by a series of structural mechanisms in society that have contributed to the establishment and maintenance of the discriminatory popular belief that Black individuals lack the aptitude for tasks with high intellectual demands. Such mechanisms reinforce discriminatory practices directed at Black individuals who have restricted access, hinder progress, and undermine self-confidence. However, these practices are not easily identifiable, as they are not always visible, which complicates the fight against this type of prejudice, known as structural racism, that impedes or hinders Black individuals access to leadership roles while maintaining the privileges of certain groups, such as White men (Almeida, 2019).

But it is not only race that is a target of structural oppression; other social markers, such as gender, serve as justifications for the discrimination of certain groups. This means that the interaction between the characteristics that make up a person’s identity (i.e., intersectionality), Crenshaw (2002) such as being a woman and Black, can exacerbate the violence suffered by the victim, making these characteristics inseparable; they must always be considered together. These factors contribute to the scarcity of Black women in leadership positions, in addition to imposing extra layers of violence that make their professional journeys more arduous (Ribeiro, 2016). Therefore, Black individuals who somehow manage to attain such positions likely arrive there after experiencing repeated acts of violence, which exacerbates the injustice, as they must deal with emotional pressures, for example, that compound the existing pressures and demands of these roles.

Specifically, sports—a sociocultural phenomenon of great magnitude, with diverse meanings and present in various forms in most people’s lives (Machado et al., 2015)—constitute a field of political dispute where various power relations are reproduced, including the dominance of a predominantly White class over racialized and marginalized Black bodies (Ferreira Junior, 2021). In the Brazilian sports scene, basketball has a long and significant history on the international stage, having won numerous titles with both the women’s and men’s national teams (Galatti et al., 2021). To promote Brazilian basketball nationally, the National Basketball League was established in 2008, responsible for organizing the men’s adult national basketball championship, named the Novo Basquete Brasil (NBB) (Meneses et al., 2016). The same occurred for women’s basketball with the launch of the Women’s Basketball League (Liga de Basquete Feminino—LBF) in 2010 (Galatti et al., 2021), promoting greater visibility through media and television channels, better working conditions for athletes and coaches, and increasing competitiveness among teams (Bemeli, 2018).

Both in the NBB and the LBF, we observe a significant number of Black athletes compared to the number of non-Black athletes. However, this pattern does not extend to leadership positions, specifically in the role of coaches, similar to what is observed in the National Basketball Association—NBA and in the Women’s Basketball Association—WNBA (Hindman et al., 2022). For this research, we analyzed the websites of the federation, the National Basketball League, news articles, and interviews about the coaches who led the teams in these leagues. We found that in all the years of the NBB and LBF up to 2022, out of 109 coaches, only 8 were Black. In 2023, there was only one Black coach in the NBB (Duarte, 2023), indicating that in basketball, the number of Black individuals in leadership positions may be lower than in the broader Brazilian professional landscape. In accordance with previous studies, we used a binary race variable, which is based on examinations of available media sources (Foreman and Turick, 2021). For many athletes becoming a coach is a common path after retiring from their athlete career, as evidenced by the number of coaches who were former professional athletes. Thus, this low number of Black coaches may indicate a hostile scenario for Black athletes transitioning to a coach career. Understanding the trajectories of Black coaches who have served or currently serve as head coaches in NBB and LBF teams can contribute to proposals actions that make the paths of current and future generations of Black coaches easier and fairer.

This study aims to investigate the perceptions of former or current Black head coaches in basketball from the NBB and/or LBF regarding the effects of racial and/or gender prejudice on their professional journeys.

## 2 Materials and methods

### 2.1 Procedures

In this study, semi-structured interviews were conducted with two Black male coaches and two Black female coaches who worked in the main Brazilian professional basketball leagues, the National Basketball League (LNB - Liga Nacional de Basquete), that organizes the men’s national championship - NBB (New Basketball Brazil), and the Women’s Basketball League (LBF - Liga de Basquete Feminino), which organizes the women’s national championship. The coaches were identified on the LNB and LBF websites., which contain information about all the teams participating in the championship editions, that is, from 2008 to 2022 for the LNB, and from 2010 to 2022 for the LBF (National Basketball League, n.d. Women’s Basketball League, n.d.). After identifying each team coach, we gathered photos of them from 52 different websites, including social media platforms in order to carry out the heteroclassification, which consists of the classification of someone’s skin color or race by a third person. The description is presented in Table 1.

**Table 1:** Number of men and women holding the position of head coach in the NBB and LBF leagues.

	NBB		LBF	
	Men	Women	Men	Women
Black coaches	3	0	2	3
White coaches	66	0	30	5
Total	69	0	32	8
General total	109			

NBB, Novo Basquete Brasil (New Basketball Brazil); LBF, Liga de Basquete Feminino (Womens’ Basketball League).

We identified a total of 109 coaches, of whom eight were Black. In the NBB, there were 69 male coaches, three Black and 66 White, while in the LBF, we found 40 coaches, both male and female, 35 White and five Black. We tried to contact all eight coaches, four replied and agreed to participate in the research, two men and two women. We understand that this is a small sample in absolute numbers, however, it represents 50% of the total population of Black coaches in the leagues studied and, therefore, the perceptions of these volunteers can contribute to the understanding of the phenomenon.

The interview guide included questions about career trajectory, the people and events that influenced their careers, how they reached their current positions, and their perspectives on racism throughout this trajectory. It was pilot tested with a 59-years-old male Black coach who worked with grassroots sport and its final version demonstrating the questions that started the conversation is provided as Supplementary material. It is worth noting that we redirected questions according to the conversation flow and made it comfortable for coaches to talk about topics not initially suggested.

The included participants completed a questionnaire specifically designed for this research to collect sociodemographic data for the description of the participants. Afterward, we conducted semi-structured interviews via a digital platform, and both video and audio were recorded for later transcription. All data were collected in 2022, the final year of analysis.

The interviews were conducted by two authors of this study, both Black women with prior experience as basketball athletes and coaches. At the time of the interviews, one was an undergraduate research student, and the other was pursuing her doctorate. The third author, a White woman with a PhD and extensive experience in research of this nature, was involved in the entire design of the study and the interview script, but not in data collection. This approach aimed to create a safer environment, exclusively composed of Black individuals, to encourage the emergence of topics experienced by Black people.

### 2.2 Participants

The names of the participants in this research are kept confidential, and pseudonyms were used throughout the study.

Ana, born in the southern region of Brazil, is a 41-year-old female coach who worked in the LBF for 2 years. She holds degrees in physical education and pedagogy and is currently working with youth teams. Ana had a 14-year career as an athlete and became a coach immediately after retiring at the age of 28.

Camila, a foreigner born in a Central American country, is 50 years old and coached in the LBF for 5 years. Camila earned many titles as an athlete, both with her national team and with teams in Brazil, where she began playing at the age of 20. She has a degree in physical education and took on a coaching role with youth teams at the club where she played, immediately after retiring as an athlete.

José is a 47-year-old Black male coach with a degree in physical education. He has been coaching in the main Brazilian men’s basketball championship, the NBB, for 6 years, and has a total of 9 years of experience as a coach, working at all levels, from youth development to elite performance. He was born in the southeastern region of Brazil and ended his athletic career at the age of 38, beginning his coaching career shortly thereafter.

Carlos was born in the southeastern region of Brazil and is 62 years old. He has coached 4 teams in the NBB, starting his coaching career at the age of 41 after retiring as an athlete. He holds a degree in physical education.

This project was approved by the Ethics and Research Committee of the University of Campinas (number omitted for blinding). All participants signed the Free and Informed Consent Form (TCLE) before data collection began. All volunteers were informed about the potential risks of participating in this research, such as the emotional distress that could arise from discussing experiences of racism, as well as the psychological support available to them if needed, in accordance with the guidelines established by the Brazilian National Research Ethics Commission (Comissão Nacional de Ética em Pesquisa—CONEP).

### 2.3 Data analysis

The questionnaires were analysed descriptively. The interviews were transcribed verbatim, and their content was checked with the participants. We analysed the data following the six steps of reflexive thematic analysis (i.e., 1—familiarization; 2—coding; 3—generating themes; 4—reviewing themes; 5—defining and naming themes; and 6—reporting) (Braun and Clarke, 2019). An inductive/deductive analysis was conducted, considering the lack of studies on the development of Black coaches, particularly in South America, and we relied on more established theories regarding racial and gender biases to identify potential patterns in the results. Supplementary Figure S1 represents the analysis process carried out by the three researchers.

## 3 Results

**Figure 1:**
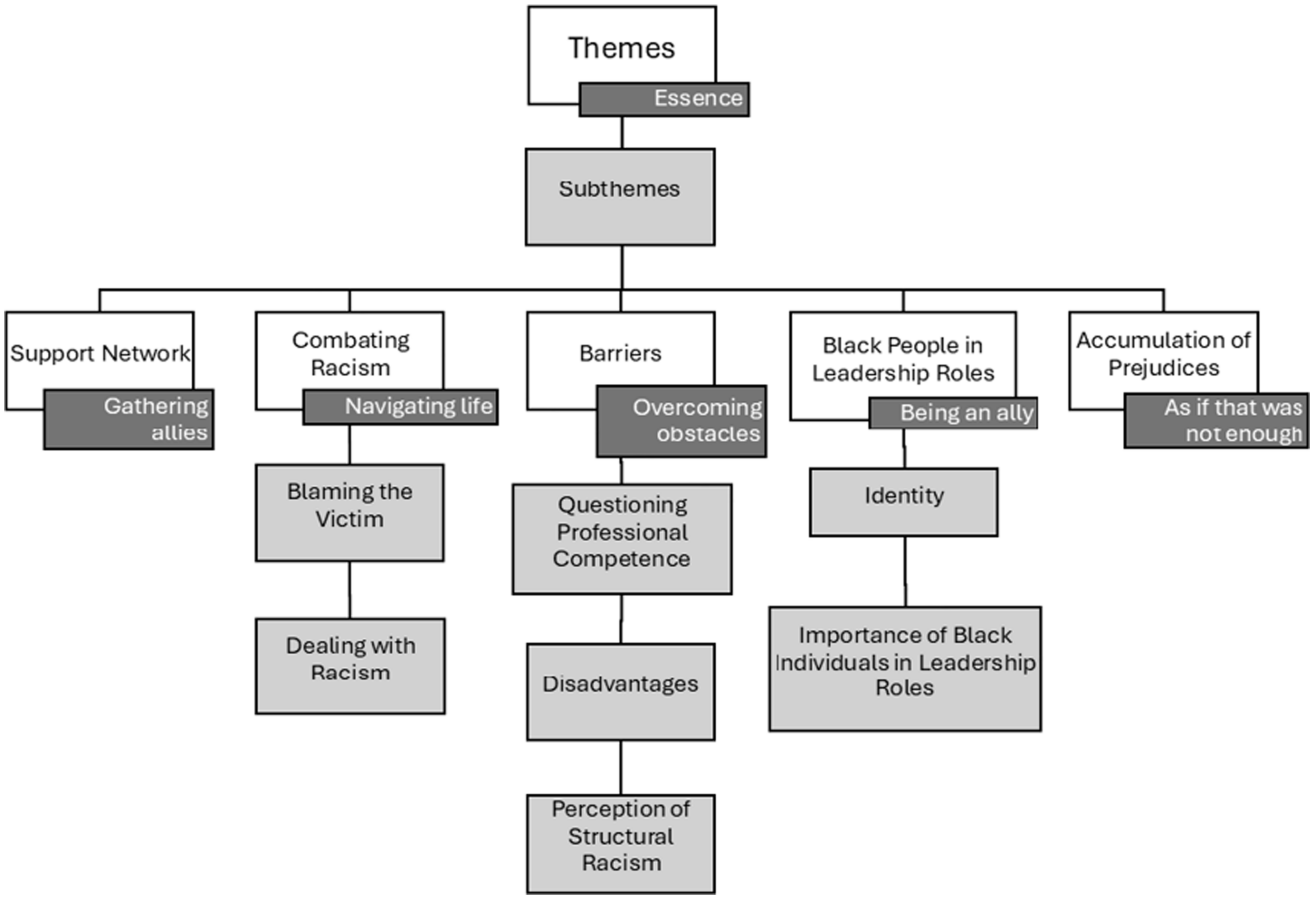
Schematic representation of the main findings. White boxes = the five themes; Smaller dark-gray boxes = main idea of each theme; light-gray boxes = seven subthemes.

The analysis of the interview transcripts resulted in 5 overarching themes and 7 sub themes that depict the main perceived barriers and supports throughout these individuals’ career paths, as well as how they dealt with the prejudice they faced, building successful professional trajectories despite challenges not experienced by their White colleagues. In this section, we present the role of the support networks the coaches developed along the way, the strategies they built to combat racism, the barriers they had to overcome related to the various forms of prejudice they encountered, the meaning they attach to seeing more Black people in leadership roles in sports, and the negative effects they observed when different social markers of prejudice intersect. Figure 1 is a schematic representation of the main findings.

### 3.1 Support network

In this theme, the coaches describe how various people contributed to their journeys toward becoming head coaches in elite basketball teams and how they felt that, without these partnerships, they either would not have reached those positions or would have felt less secure on the path. Although everyone needs support in their professional journeys, these coaches perceived the support they received as essential to diminishing the effects of structural racism on their careers, which they understood as an additional obstacle on their path.

The interviewees explain how their careers started, noting the influence of people who already knew them from their previous athletic careers and trusted their abilities enough to open doors for them. The coaches believe that the opportunity to work at a high-performance level in a leadership role would not have happened without the support of these individuals, as José explains: “I think in my career, what I did on the court and the relationships I built on the court helped me get the opportunities.” José refers to the opportunity he had to become head coach of Club A early in his career, where he began his basketball journey as an athlete. He believes this was the chance to showcase his work as a coach, which was facilitated by the relationships he had built within the club.

“And the leadership role, the opportunity I had to be head coach, came from Club A, the team where I was trained as an athlete, you know? In the neighborhood where I was born, at the club where I grew up, a club where I won many titles as a player. So, I had all this goodwill to come back and show my work, you know? So, my opportunity was given mainly because of what I had built on the court, you understand? And it was in a place where people already knew me, and I think if it hadn’t happened that way, my first step would have been different because after that, I did well, I showed results, and so on. I managed to break into the market, you know?” (José)

Coach Ana also comments on the people she met during her athletic career and on completing her degree in physical education, which allowed her the opportunity to work as a basketball coach for children and as an assistant coach, giving her the chance to experience a coaching role before taking on the head coach position.

“[…] Maria was playing and had a basketball school, and she invited me to work there. I was still a junior player, but I enjoyed being around the kids. I think I already had that coach instinct. So, she invited me to help, and I was an assistant, helping in the school. Later, when I moved to other clubs and ended up in City X, which was the last club where I played, I finished my degree in Physical Education. At the time, Jorge was our coach, and I was already assisting him. I was a player and an assistant. I wore the uniform, but I was assisting, and I started enjoying it.” (Ana)

Carlos shares how he got his first opportunity to lead a team and emphasizes the importance of seizing the moment when it arises, as it might not come again. The current head coach, who Carlos would eventually replace, had promised him the position once the season ended. However, when the coach realized he would not be able to finish the season, he offered the position to Carlos right away, knowing that Carlos would not have had the chance otherwise.

“It had to be quick; the decision had to be quick. I had initial doubts, but then this coach friend of mine said, ‘Imagine if you stay here until the season ends as a player. Then a new coach comes in, resumes the work, and who knows if you’ll still be here or if he’ll want you? The plans we had for you to be my assistant coach won’t work because I’m leaving, but you can be the head coach.’ And I thought, ‘He’s right,’ and I changed course, accepted the offer, and stopped playing.” (Carlos)

Another point raised was the importance of support networks for these coaches to achieve their positions. For Ana, the encouragement and trust of certain people, even when she doubted herself, were crucial.

“Maria said, ‘Ana, we need you to take over the team because Jorge can’t do it right now. We need someone who represents basketball here in City X, and that person is you! Can we do this together?’ And I remember saying to her, ‘But I love the kids, what about the kids?’ And she said, ‘Don’t worry, it’ll all work out; we’ll make it happen.’” (Ana)

For Carlos, the support network played an essential role in his career. He realized that his skills and knowledge alone were not enough to keep him in the position; he needed the trust of others.

“And I stayed at Club B for 7 years, always backed by someone. It’s interesting because sometimes it’s not just about your ability, your knowledge, or your dedication. No, it’s also about your friendship with someone, or whether someone likes you more or less, and that person has the power to move you around, keep you here, or take you away. So, these things happen.” (Carlos)

The support from close friends and family also played a crucial role in Ana’s career. The encouragement to persevere and be resilient in following the path she had chosen, as well as the support to finish her degree, gave her the strength to continue her journey.

“My mom always said, ‘It’s very important for you to have a profession; it’s very important for you to have a degree,’ so my mom encouraged me, and my uncle, who was already a Physical Education teacher, encouraged me as well.” (Ana)

### 3.2 Combating racism

In this theme, the coaches describe their perceptions of the subtle racism they have experienced throughout their lives and careers, as well as the strategies they developed to combat the effects of this prejudice, such as victim-blaming. This is a commonly used and widely disseminated strategy to normalize prejudice, exonerating the true culprits. They also discussed the individual strategies they found helpful in dealing with the racism they faced.

#### 3.2.1 Blaming the victim

In this subtheme, we highlight the coaches’ narratives about how they used to justify the racism they experienced by blaming themselves or victims of prejudice in general. This strategy can be considered dysfunctional, as it can increase the victim’s suffering while absolving the aggressor. By blaming themselves for the racism they experienced, they gained a sense of control over their own lives, giving a false sense of empowerment. This logic assumes that if they are to blame, they can somehow change the situation by taking individual action. However, while this strategy might have some impact on the individual trajectories of each coach, the structural aspects that hinder their professional journeys remain invisible and unchallenged, perpetuating this kind of prejudice. It’s clear that the discomfort victims feel when confronting structural racism is significant, given the sense of powerlessness against the violence unacknowledged by society and the invisibility of their suffering. Thus, this strategy can be understood as a defense mechanism against this pain.

For example, when asked about why we see so many Black athletes but few Black coaches, Camila initially attributes this to the individual motivations of each athlete, who may choose to follow other paths. While her statement is true, it simplifies a complex phenomenon.

“So, as I mentioned, it depends on the person, it depends on whether a Black person wants to choose this career. In my case, I’ve been a player since I was 8 years old, so I chose physical education as a profession. There are people who don’t identify with it. In my case, I pursued it because I wanted to be a coach, but I encountered a lot of difficulties, you know?” (Camila)

When asked to give advice to hypothetical Black coaches aspiring to reach his position, Carlos emphasized the need for self-improvement, presenting a solution based on individual actions that worked for him and therefore are validated by his experience. However, considering the low number of Black coaches in Carlos’s context, we can conclude that while this strategy may have localized success and is necessary for professional development, it does not reflect structural changes that could help more black coaches follow this path. Additionally, the logic of individual responsibility is evident, implying that as long as one acquires skills, failing to achieve the goal may be blamed on a lack of effort in pursuing professional development courses, for example.

“I think self-improvement is key. You need to watch a lot of games, find references you identify with, and try to get as close as possible to them to understand their process. I think the advice I would give is to self-improve to have clarity in your steps. And then, don’t be rigid—being open to new ideas is important—but I think you need to have an identity. And I think you can build that identity as you develop yourself. In the end, it’s all about education and learning. I believe studying is the way.” (Carlos)

#### 3.2.2 Dealing with racism

In this subtheme, the coaches describe strategies they adopted to minimize the effects of racism in their professional careers and personal lives. José expresses his surprise and discomfort at being considered for and occupying a position that society does not usually associate with black people. For José, the comfort zone was never being considered for the role he aspired to.

“[…] I was always overlooked, and now I’m not overlooked anymore; I’m chosen. And then I say, ‘Wow, this feels weird.’ You’re not comfortable with it, even though it’s a good thing. You go from being overlooked to being chosen, and that’s good. Now they’re choosing me, but because I’ve been overlooked so much, it creates anxiety and discomfort. My comfort zone, what I was used to, was being overlooked. So, it’s like that—sometimes we’ve taken so many hits that being in the comfort zone feels normal, it’s normal, it’s normal.” (José)

Comparing his professional journey to that of non-Black coaches, Carlos comments on how the absence of Black people in coaching staff positions is normalized.

“How can I put it? It’s something so normal for us, so everyday, that people don’t even notice it. If we don’t talk about it—because sometimes we remain silent, and even I wouldn’t have talked about this if you hadn’t asked for this interview. I wouldn’t be discussing this, which is our fault too, so we don’t talk about it. But we need to start diversifying.” (Carlos)

Frustrated with the normalization of this phenomenon, Carlos questioned a non-Black colleague about his ability to act in favor of changing this scenario, but with no success.

“I said, ‘Marcos, why can’t you change this? For example, in your team, why not have a Black person on the coaching staff? Have you noticed that there’s not one on the staff?’ He thought for a moment and said, ‘Yeah, you’re right.’ But it wasn’t his cause, you know? It wasn’t his issue.” (Carlos)

The experience of understanding the social structures that hinder their professional paths helped these coaches develop strategies to keep pursuing their goals. José comments on this: “When the tie benefits the other team, it’s always the other’s. So, you always have to play to win. It’s complicated because I’m in this limbo of ‘it’s good and it’s bad.’

Reflecting on the changes she observes in society, Ana acknowledges that racism still exists, but due to social pressure, it’s less explicit — people do not speak openly about it, and they feel pressured to moderate their behavior when addressing Black individuals. When she says, “[…] they are being careful, not changing the way they think, but being careful about how they speak about Black people, especially Black women,” Ana recognizes that racism and sexism aren’t directly targeted at her, which she views as progress.

Another strategy to minimize violence was always demonstrating kindness, being very polite, and making an effort to be seen as a pleasant person, especially in challenging or vulnerable situations, with the hope of advancing professionally by earning the sympathy of those who make career-influencing decisions.

“Oh, because you’re Black,’ I may be Black, but look, ‘I’m better than you because I do this, I do that, and you don’t, you can’t do it.’ So be polite when facing someone who challenges you, be classy, rise above.” (Camila)

“[…] because I’m always polite—I’ve always been very polite on and off the court. I’d show up, but I didn’t talk much, and maybe that wasn’t the best approach. Being that way wasn’t… it’s just who I am, you know? But it didn’t help much because if I had been more flexible, more talkative, and smiled more, maybe I would’ve come across as more pleasant.” (Carlos)

The strategies adopted by these coaches do not change the racist structures that create the challenges they face. Still, they represent individual approaches to avoiding further harm, such as the effort to always appear pleasant.

### 3.3 Barriers

In this section, the coaches describe the obstacles that have made their professional journey more difficult or convoluted. These barriers have been overcome through strategies developed over the course of their careers and personal lives, yet they still add additional steps to these individuals’ paths. Constantly having their professional competencies questioned, they feel the need to be better prepared than their peers in order to provide convincing arguments. Moreover, they perceive themselves as starting from a disadvantage, as if the race toward their goals is longer for them. In trying to make sense of this, they recognize a social structure that resembles a force pulling them in the opposite direction of the path they have chosen to follow.

#### 3.3.1 Questioning professional competence

The coaches report being continually questioned about their ability to perform their roles. They feel a persistent need to prove their effectiveness and perceive a low tolerance for errors, which contrasts with the treatment received by non-Black individuals. José describes the pressure he feels when he has a professional opportunity: “So, I think that’s it. When a Black person gets a position, it’s like having a knife at their throat; they have to prove themselves. There is not the luxury of ‘let us work with you,’ you know?”

While other professionals might focus on preparing to excel in their work, José grapples with the need to feel more prepared in order not to be excluded: “[…] but in my process, I often felt I had to please everyone, that I had to be exceptional because if I wasn’t or did not put in the effort, I would be excluded […].” The fears of these coaches are realized, as Carlos describes: “I worked in City X, I won more than I lost, and I was fired because I lost a game there, and I was fired. It was just, I do not know, whatever, I was fired,” indicating that he was not informed of the reason for his dismissal despite a record of victories, which are highly valued in decisions regarding the retention of coaches in sports, in Brazil at least.

Another point raised by José illustrates how he perceives that Black individuals are often praised only for their physical attributes when it comes to roles requiring physical strength, expressing dissatisfaction with the scrutiny he faces while attempting to fulfill a position that demands high intellectual capacity: “Because when it comes to athletes, our physical attributes, which are theoretically superior—strength, power, and so on—come into play. But regarding the intellectual aspect, knowledge, etc., we are questioned.”

Another expression of this excessive questioning is the low tolerance for error that these coaches experience while performing their roles, a behavior not observed towards White coaches, creating tension among Black coaches, as explained by José and Ana:

“[…] I feel that if I were to perform poorly in that first year, I would easily be labeled and left behind, you know? What I feel is that the tolerance is much lower, you see? The ease of labeling a Black person's lack of success in a leadership position is much quicker than for a White person.” (José)

“It was a big challenge, and the expectations were high for this team. In the first game, we lost by 20 points. Can you imagine everything that went through my mind? Everything that went through the directors' minds? I think they considered firing me.” (Camila)

Thus, these coaches face an additional layer of pressure and demands that not only add tension but also require an effort not experienced by White coaches.

#### 3.3.2 Disadvantages

In this section, the coaches describe how they feel disadvantaged in their professional trajectories, facing fewer opportunities even when they are prepared. José comments on the obstacles faced by Black individuals and the injustice of not being able to compete professionally under the same conditions as others:

“What I believe, and I often tell my friends when we discuss this, is that being Black doesn’t mean you won’t run the same race; you will run the same race, but you start behind. It’s like we are running a 100-meter race, and the Black person starts 100 meters behind.” (José)

“I wanted more based on my ability and commitment; I wanted more because I have a lot to offer, but the opportunities don’t open up. But I don’t martyr myself over it. I start to think, ‘Well, the bad luck isn’t mine; it’s theirs,’ as frustrated as I may be with all this. And maybe I’m just like everyone else; I stay silent, and sometimes we don’t have the opportunity to speak about it.” (Carlos)

Carlos describes his frustration with the lack of opportunities and the silencing he experiences. The feeling of always being behind is constant, and these coaches face the routine demand to “prove their worth” in order to secure the opportunities they believe they already deserve.

“But what I see is that a Black person always has to pay first to then have the opportunity, you know? You always have to prove you are better to get the opportunity. If you are equal, the opportunity is often given to others, to those with White skin.” (José)

Another observed disadvantage is the homogenous reproduction within the field, as Ana explains, which she considers a disadvantage since most managers and leaders are White males (Foreman and Turick, 2021).

“So sometimes there are people who end up leaving this profession where they could excel, perhaps out of fear of what could happen and what they might face ahead. All of this can weigh heavily on people. But I imagine that this tendency to give opportunities to those who are already closer is what generally prevails.” (Ana)

This phenomenon is echoed by Camila, who explains that she received an opportunity due to an invitation from a woman. However, she highlights her potential to reach higher positions, consistent with her achievements as a coach in her current and previous career, as an athlete.

“And I was never given any opportunity; the opportunity I’m getting now is from this woman who invited me to be a coach, the only one who valued me. Here in my city, I’m a sports initiation coach, but I could be a project coordinator. I could be something bigger, but no one asks, no one speaks; they have implemented various initiatives but don’t care at all. So, being a woman and Black is already difficult, and being a foreigner makes it even harder.” (Camila)

#### 3.3.3 Perception of structural racism

In this subtheme, the coaches report their perceptions of a racist social structure that hinders their professional trajectories. Being structural, it is often imperceptible to many, as its effects are naturalized. Consequently, the actions taken by the interviewees are often discredited, as José expresses: “It’s difficult for me to speak because racism in Brazil is always very veiled. So, when you talk about it, you are always met with ‘Oh, you are exaggerating,’ ‘Oh, it’s not like that,’ ‘Oh, it’s not such and such.’” Ana no longer works in the field and points out that it is common for people experiencing this type of prejudice not to express their discontent or complaints:

“I think that many times I didn’t hear about the Black experience because maybe the person thought it but didn’t say it, knowing that nowadays such behavior is not well accepted. We don’t accept it; it’s not that we used to; we kept quiet, we were afraid of the consequences, and now we aren’t afraid anymore.” (Ana)

Carlos’ comments illustrate the silencing and the normalization of a mindset among those in leadership positions in Brazilian basketball that establishes mechanisms that exclude Black individuals from these roles.

“Yeah, because for me it hasn’t been explicit yet. […] There are plenty of competent people, so why aren’t they in those layers of the league, of the CBB [Brazilian Basketball Confederation], that kind of thing? If this voice isn’t heard, isn’t spoken about, if you accept it, and as I told you, it seems like… I don’t think it’s normal, but I don’t know the term that fits better; it’s something that’s there, we’re already anesthetized to it.” (Carlos)

The interviewees also highlighted another expression of structural racism, relating to the fact that while there are many Black athletes, many considered outstanding, few transition to coaching careers, as José describes:

“And in sports, you don’t need to look very hard; just look at the coaching staff, the basketball teams; just look at the coaching staff in volleyball and soccer. I can count them on one hand. How is it that we are the majority, yet we are the minority in coaching roles?” (José)

José further expresses his discontent by pointing out the absence of Black individuals in other roles within coaching staff:

“It’s because I’ve known it since I was a child.’ That’s how things have always been, and they follow that path. It’s no wonder that here I think I’m the only Black coach in the NBB; I think I am the only Black coach, and very few others are assistants or physical trainers; the number is infinitesimal. If you think about it, it’s very small. Why is that? ‘Oh, they lack ability, training?” (José)

Ana describes how achieving the position of coach did not guarantee that she was considered competent or deserving of retention in that role:

“[…] but there were opportunities for me to be there through my work, grassroots basketball, a structure built over many years, working hard, trying, fighting, and then someone comes in and says, ‘I want to be here, doing this, this, and that,’ and the people above me simply say, ‘Now you step aside because this person is coming in,’ and this person was White, this person was a man.” (Ana)

Thus, the frustration of these coaches is evident as they recognize their competence while grappling with the challenges they face in achieving their objectives, as José points out: “How is it that we are the majority, yet we are not the majority anywhere—not in one place or another, nowhere, in no sport, you know?”

### 3.4 Black people in leadership roles

In this section, the coaches recognize their identities and describe how they see themselves in the world. Ana explains, “Look, I’m going to tell you, this is a very difficult topic to discuss. I have always considered myself a Black person, being the daughter of a Black father and a White mother; I have always seen myself as Black.” Based on their life experiences, they highlight the importance of Black individuals holding leadership positions, such as those they have occupied as coaches.

#### 3.4.1 Identity

In this subtheme, the coaches describe how they perceive themselves and how they constructed their identities. In the sociodemographic questionnaire, José identifies as light-skinned Black. At the beginning of the interview, when asked to confirm this information, José states, “I have complete awareness of my Blackness, so to speak. After all, my entire family, on both my mother’s and father’s sides, are all Black. I consider myself Black; the light-skinned Black label is just for the certificate.” José refers to the fact that on his birth certificate he is described as light-skinned black. Ana, a light-skinned Black woman, recounts the discomfort she has felt throughout her life when others constantly tried to convince her that she was not Black.

“But over time, some people couldn’t convince me because I believe we have our own personality and opinions. However, many people criticized me for saying I was Black, saying ‘No, you’re not Black. Really? Look at you; you’re light-skinned, you’re ‘sarará’ [an informal term used in Brazil to describe light-skinned Black people].’ That’s what people would say.” (Ana)

Camila also reflects on the meanings she assigns to her identity as a Black woman:

“Being Black is also a privilege; it’s a privilege because you are unique, and you can show the world that being Black means you can be better, much better than others think. So, for me, being a woman and Black is the greatest challenge and the greatest privilege I feel. To me, it embodies both a challenge and a privilege.” (Camila)

By mentioning that being a Black woman is both a privilege and a challenge, Camila demonstrates an awareness of the difficulties she has faced and continues to face, while also appreciating herself.

“So, it’s about being careful not to try to fit into a role that isn’t ours, seeking acceptance, you know? ‘Oh, I’m not accepted, so I’ll mold myself, I’ll try to fit in. I’m salt, but I want to be sugar.’ If you’re salt, you’re salt; that’s it.” (José)

Like José, the interviewees show awareness of who they are, acknowledging their life experiences related to their Black identities and how this connects to their chosen profession, shaping the relationships they establish in their professional context.

#### 3.4.2 Importance of Black individuals in leadership roles

The coaches recognize the importance of having more Black individuals in leadership positions and see this as a crucial point for changing the current landscape. José emphasizes his commitment to providing development opportunities for other Black individuals, revealing his awareness of the disparities in opportunities available to Black and non-Black individuals.

“Ah, my coaching staff is the most Black in the NBB [laughs]. Not because it’s forced, but I see the importance of saying, ‘Hey, come here and develop just like others can.’ I have no prejudice; I do open opportunities for them to experience this and grow within it.” (José)

José and Ana describe their sense of responsibility to contribute to the paths of future generations, facilitating a process that was more challenging for them.

“I believe we also need to be committed to and develop others, right? If I can take a step forward, I should look back and extend my hand so that others can also move forward. If I can climb the mountain, I should throw a rope to help someone else climb it too. I think this will create a virtuous cycle for change in the medium and long term.” (José)

“Because it opens space for other people and changes the mindset. Sometimes, as I told you, a young woman might think she isn’t capable and wonder, ‘Am I going to go there and then hear so much nonsense? Why should I enter this? I might end up hurt; forget it. I’ll do something else and leave this aside.’ So, the more Black women in leadership, the better for us. It provides more encouragement for those coming up and for those growing up, showing that things need to be different, that things must change. The more, the better, for sure.” (Ana)

Ana and Camila describe the importance they place on being positive role models for athletes, encouraging development, and showing their willingness to help. Yet, they also express vulnerability regarding the futures of young people in Brazil.

“I have been able to support people, boys and girls who have gone through my programs and are now graduates. I would tell them, ‘Guys, go to college! In whatever way I can help you, I’m here to help.’ There’s always been that interaction with the sports department, and we’d say, ‘Look, there’s a student who is applying for college and got in. Let’s get a scholarship,’ and we were able to do that. Thank God, just as I received support, I was also able to encourage many of my former athletes to graduate and pursue careers as physical education teachers.” (Ana)

“I want to create courses for coaches, especially Black women coaches. Everyone can participate, but the main goal is for many Black individuals to take part in this project. I want to teach courses on how to introduce basketball, how they should approach these kids, how to integrate basketball into their lives so they fall in love with the sport. That’s my project; I want to set up these courses to reach out to these individuals and attract more of the Black community.” (Camila)

### 3.5 Accumulation of prejudices

The intersection of prejudices was very present in the statements of the coaches. The women coaches emphasize the challenges they have faced in aspiring to work professionally in a predominantly male environment. Ana expresses her dissatisfaction while also showing self-doubt when stating that “women deserve to be considered, even as women.”

“So unfortunately, it’s that thing where we have to prove every day that, no matter how much we are women, we have the capability to be at the top as coaches or in any other profession. I just wish we didn’t have to prove this every day; I wish people would look and say, ‘She is a woman, but we will give her a vote of confidence.’” (Ana)

The female coaches describe their confusion in not being able to determine whether the prejudices they face are due to being black or being women.

“[…] since I have only worked with men and not with women, I think the story I told back at Location X where I had that impasse, I don’t know if it was because of the color of my skin, but I know I suffered a lot, and I think it was mainly because I am a woman, not just because of my skin color but because I am a woman.” (Camila)

“So sometimes we think, ‘Wow, is it really that, or is it better not to think about it, better to delete it from my mind that it was that, better to find another excuse, like, is it just because I’m a woman, or is it because I’m just younger, or is it because I don’t have much experience? Should I just leave it at that? Or should I consider that what weighed in was racism?’” (Ana)

“During the course of this championship, I faced many difficulties; I brought in an assistant coach who started to undermine me, and I had to fight against that, you know? It was very difficult, and yet, they believed in me, and it was the biggest challenge of my career because I was black, a woman, and a foreigner, so I was not well regarded.” (Camila)

In addition, José has a condition that causes a change in the color of his skin and recounts the difficulties he has faced as a result: “Because the condition has this characteristic, it is much more visible on black skin than on white skin; it draws much more attention and is much more exposed than on white skin.”

“As a coach, I have experienced situations where people do not greet me because they are reluctant to shake my hand. It’s sad; I believed more in the ignorance of the person than in anything else. But it’s more about the person wanting to protect themselves from an imminent contagion than anything else.” (José)

In general, the accumulation of different social markers that are targets of prejudice in our society has exacerbated the suffering of these coaches, imposing on them demands that non-black coaches typically do not face.

## 4 Discussion

The results of this study contribute to the understanding of the barriers and supports that Black basketball coaches face on their journeys to elite basketball leagues in Brazil. It also enhances the comprehension of the human strengths, resilience, and strategies that these professionals develop to achieve success in their careers. Generally, the coaches experienced specific challenges in their professional paths that made their journey more difficult compared to their white counterparts. The extra effort they had to expend was recognized by these coaches, and after reaching the position of head coach in their teams, they look around and find themselves nearly alone. They then take on the responsibility of paving the way for a new generation of coaches like themselves, trying to ease the journey for those following the same path (Foreman and Turick, 2021).

Taking on the role of head coach was not an easy task for the participants in this research, as corroborated by the accounts presented, and a solid support network in sports and their personal lives was a decisive factor. The coaches noted that the relationships they established while they were athletes enabled their entry into coaching, as it was generally their former coaches who invited them to begin their journey. A notable characteristic in the careers of coaches is the need to establish a network of contacts that allows for entry and continuity in this field. This, combined with structural racism, which excludes Black individuals from management and leadership positions by perpetuating the belief in their inadequacy for such roles and offering decision-making positions to white individuals (Fast and Jensen, 2006 Cunningham, 2020 Foreman and Turick, 2021), makes the journey of Black coaches more challenging, with an exacerbation in the case of Black women. This occurs because there is a tendency for white coaches to endorse the hiring of other white coaches (i.e., homologous reproduction—Kanter, 1977). Thus, relying on a close network of contacts to start a career can be complicated for most Black individuals wishing to become coaches, as the criteria and minimum competencies required for the position remain subjective and unclear, hindering their preparation for the role.

The support these coaches received from their families was also crucial. Emotional support and understanding were highlighted as decisive factors. The coaching career in professional leagues is challenging. In addition to uncertainties, coaches must deal with constant travel, time away from family, inadequate salaries, frequent job changes, among other issues (Carson et al., 2018). For the interviewed coaches, sharing uncertainties, receiving emotional support, and encouragement were very important in the decisions they had to make throughout their careers, such as relocating. Seeking social support by Black individuals who are victims of racism is an important behavior that can help mitigate the negative impacts of racism on the well-being of Black people (Marshburn and Campos, 2021). Thus, considering that the stress and insecurity faced by Black coaches throughout their careers may be exacerbated by structural racism, emotional support networks play an essential role in their professional journeys.

The strategies that the interviewed coaches developed to confront the racism they experienced throughout their professional paths proved effective for each of them, allowing them to achieve their goals despite suffering. However, considering that racism is part of an oppressive structure that establishes a power dynamic in which Black coaches have little or no power (Almeida, 2019; Rankin-Wright et al., 2017), the strategies employed by these coaches, emphasized as necessary for future Black coaches to enter the system, tend to be dysfunctional for most individuals and may cause further suffering. Being motivated to pursue a career as a coach in elite basketball does not guarantee success for anyone, regardless of skin color, but the chances are lower for racialized men and even lower for racialized women. Additionally, when they reach the position, recognition and respect will be lesser compared to their non-Black peers (Rankin-Wright et al., 2017). For instance, Obenauer and Langer (2019) analyzed data on the performance of coaches and teams in the National Basketball Association and found that Black coaches who had experienced an early period of success in their career were more likely to be fired and less likely to receive awards then White coaches. Therefore, the coaches participating in this study, who are situated in a society whose dominant culture marginalizes Black individuals (Silva et al., 2021), express legitimate ways of thinking, as they are a social construct, but these thoughts reinforce the perpetuation of structural racism, maintaining the privileges and power of certain groups over others, as the focus remains on the victim and the social structure is not questioned (Nunes, 2010).

The normalization of occupying more socially recognized positions with greater decision-making demands by White men legitimizes discrimination within organizations, including in sports. This ensures that discrimination is no longer interpreted as harmful or offensive by individuals, and thus it goes unnoticed, is reproduced, and perpetuated (Rankin-Wright et al., 2017; Almeida, 2019). Therefore, the discomfort expressed by coach José upon being selected for the head coach position can be interpreted as an internalization of the socially constructed belief that Black individuals would not be capable of fulfilling this role.

The interviewed coaches acknowledge barriers that have hindered their career advancement throughout their personal and professional lives. This study shows that there are few Black individuals who attain the position of head coach in elite teams in Brazil, at least in basketball. These coaches reported facing constant questioning of their abilities to perform their roles, which reproduces the belief that Black individuals are incapable of holding leadership positions. These factors suggest a scarcity of opportunities, and the coaches’ accounts indicate an increased emotional burden necessary for maintaining their positions. This adds an obstacle to their journeys that is not present for white coaches, for example, constituting a disadvantage (Picanço, 2014).

When studying the experiences of racialized coaches in the United Kingdom, Rankin-Wright et al. (2017) observed that racialized coaches avoided making mistakes as much as possible to prevent questioning and distrust regarding their abilities. Therefore, while non-racialized coaches focus on performing their roles well, racialized coaches face the additional demand of avoiding errors, which is contradictory since mistakes are one of the variables that prompt us to reevaluate decisions and improve. This is corroborated by Hindman et al. (2022), who analyzed data from the 1997 to 2021 seasons and found that Black women, Black men, and White women coaches in the Women’s Basketball Association were more likely to be fired than White men. Although better performance, measured by wins, reduced the likelihood of dismissal, racialized coaches and White women remained at higher risk of losing their positions even after controlling for performance. This suggests that winning more games, or making less mistakes, may not be enough to offset the impact of racial and gender biases in job security. Rankin-Wright et al. (2017) also found that the volunteers in their research attempted to downplay their identity traits by adopting behaviors of the dominant group to integrate into the context. While this strategy yields some effect, as the coaches in the mentioned study were indeed part of the context, they continue to be perceived as less competent to perform their roles, as racism structures society and their phenotypic characteristics of race and gender trigger prejudice (Almeida, 2019; Puwar, 2004). Thus, from a collective perspective, adopting behaviors of the dominant group reinforces the perpetuation of racism.

Ana’s discomfort at being categorized as white by acquaintances exemplifies the complexity of the phenomenon of identity construction and affirmation for Black individuals occupying the role of head coaches in sports teams. While assuming this classification could ease her professional life, it represents a significant burden for her, as she identifies as Black. Generally, the coaches participating in this study are confident in their identity and, being in leadership positions, can help future Black coaches see themselves on this path, in addition to opening doors for these coaches by challenging the system, questioning imposed certainties, and bringing more Black individuals into their coaching staffs, as exemplified by coach José, who asserts that his coaching staff “is the most Black” in the championship. By examining the history of the presence of Black coaches over 32 years in the American football league (NFL—National Football League), Foreman and Turick (2021) found that Black coaches in supportive roles on coaching teams, such as assistant coaches, were less likely to assume central leadership positions. However, when these coaches were led by head coaches who were part of marginalized groups, the chances of them occupying central leadership roles increased during certain periods of the research.

Openly embracing one’s identity and leading from it is not an easy task, as reaffirming one’s views on personal aesthetics, intellectual quality, and dignity as a human being represents a daily struggle. This emotional burden can be difficult to bear (Gomes, 2007). We found that the coaches interviewed in this research actively seek self-affirmation and remain vigilant about the messages they receive regarding their appearance and abilities. Their pursuit of self-appreciation demonstrates a robust sense of confidence in their competence to excel in their chosen profession. Notably, these coaches have reached a highly sought-after position, indicating that they have cultivated essential skills such as self-confidence and resilience, which have significantly contributed to their professional achievements.

In this context, the concept of intersectionality becomes particularly relevant, as it underscores the complex interplay between race and gender that shapes the identities and experiences of these coaches. Intersectionality recognizes that race and gender are not merely additive but are intertwined structural oppressions that exacerbate the challenges faced by marginalized individuals Crenshaw (2002). This is particularly evident in the experiences of Black women, who navigate the compounded effects of both racial and gender discrimination (Ribeiro, 2016). Bruening (2012) notes that Black female coaches often face distinct forms of racism within the sports environment, which differ markedly from those encountered by their Black male peers. The female coaches interviewed have highlighted the unique challenges they face in their work environments due to their gender; many express uncertainty regarding whether the prejudice they encounter stems from their gender or their race. This confusion resonates with the findings of Rankin-Wright et al. (2017), who documented similar experiences among Black female coaches.

The coaching career presents significant challenges for all women (Barreira, 2021), but Black women face additional hurdles that intensify their struggle for recognition and advancement (Campos, 2023). For instance, Camila, a successful athlete and head coach for a women’s team in the LBF, points out the scarcity of opportunities afforded to her, despite her qualifications. Her experiences illustrate how intersectionality shapes identity and professional pathways, as the dual challenges of gender and race create formidable barriers. Furthermore, intersectionality encompasses the unique experiences of individuals like coach José, who faces distinct forms of adversity tied to both his racial identity and specific physical characteristics. His experiences reflect how different axes of identity converge to create complex realities marked by multiple forms of oppression. This interplay, which compels him to navigate aggressions stemming from different sources, exemplifies the importance of understanding intersectionality as a critical framework for analyzing the lived experiences of Black individuals in professional settings.

## 5 Conclusion

The findings of this study reveal that Black coaches in the Brazilian basketball scenario still face difficulties entering the elite leagues as head coaches and, once they managed to achieve the desired position, they struggle with the lack of support and structural racism, making them feel they do not fit there. This is not new finding, but the persistence in the occurrence of these patterns of behavior indicates that we need to do a better job as a basketball community, specifically managers and decision makers, in order to offer more equitable opportunity and treatment for current and future Black coaches.

This study demonstrates the complexity of the experiences of Black basketball coaches, emphasizing the barriers and supports that have impacted their professional trajectories. Support networks, both in sports and in personal life, emerge as essential for overcoming obstacles imposed by structural racism, enabling these professionals to reach prominent positions in elite teams. The analysis highlights how these coaches build strategies to deal with prejudice, demonstrating resilience and asserting their identities. However, the still limited presence of Black coaches in leadership positions, particularly Black women, underscores the need for more opportunities and structural changes to address the intersecting challenges of racism and sexism they face.

In practical terms, these findings indicate that basketball leagues with similar characteristics to the two that were the focus of this study should promote inclusive policies to increase Black representation in leadership roles, enhancing the visibility of these professionals and enabling them to serve as role models for future generations. For example, systematic identification and monitoring of Black athletes willing to pursue a coaching career and development of coach education programs within institutions so these people have better chances to succeed. Mentorships could serve as additional support, helping to reduce inequality in access to opportunities. For example, the Brazilian Olympic Committee offered in 2024 a mentorship program for women coaches in an effort to reduce gender inequities in sports coaching. Ten elite female coaches had to opportunity to be mentored by experienced coaches for 1 year, participating in online and face to face activities. The program was called Individualized Mentorship Reflection and Action (MIRA – Brazilian Olympic Committee, 2024). This is an example of a mentoring program that could be designed and targeted at Black coaches already working in high-performance basketball who, similar to the coaches interviewed in this study, may feel isolated or professionally invalidated and could benefit from mentorship provided by more experienced colleagues and the establishment of a meaningful professional network. Additionally, the importance of personal and club-based support networks highlights the need for spaces where these coaches can share their experiences and find emotional support, a crucial factor in facing the psychological and professional impacts of racism. Sports organizations must, therefore, review institutional practices to create environments that recognize each individual’s competencies and foster their potential, regardless of race or gender. This could be achieved by educational programs targeted at sports managers and decision makers, for instance.

We highlight the small sample size in this study as an important data, a denunciation of the low number of black coaches in the main Brazilian basketball leagues. On the other hand, it prevents broad generalizations and suggests that this topic would be relevant for future research. The intersection of different social markers, such as race and gender, and their impact on leadership opportunities within sports more broadly, may also be explored in future research. Longitudinal studies on the career progression of Black coaches across various sports could provide a better understanding of changes over time and an empirical basis for structural interventions and affirmative policies. Moreover, investigations analyzing the perspectives of sports managers could shed light on institutional barriers and attitudes that perpetuate racist structures, offering valuable insights for building a fairer and more inclusive sports environment.

## Data Availability

The datasets presented in this article are not readily available because the dataset contains information that could identify the subjects. Requests to access the datasets should be directed to bartirapalma@gmail.com.
