# Corrigendum: Of the few Black coaches in Brazilian professional basketball leagues: approaches to racism

**DOI:** 10.3389/fpsyg.2025.1628813

**Published:** 2025-06-02

**Authors:** Julia Oliveira Marcelino, Bartira Pereira Palma, Larissa Rafaela Galatti

**Affiliations:** ^1^Laboratório de Estudos em Pedagogia do Esporte – LEPE, Faculdade de Ciências Aplicadas, Universidade Estadual de Campinas, Limeira, Brazil; ^2^Faculdade de Educação Física, Universidade Estadual de Campinas, Campinas, Brazil

**Keywords:** sports, prejudice, professional career, coach development, social justice

In the published article, there was an error in Table 1 as published. There was an inversion in rows 3 and 4 of the table, which incorrectly suggested that there are more Black coaches than White coaches, when in fact the opposite is true.

The corrected Table 1 and its caption appear below.

**Table 1:** Number of men and women holding the position of head coach in the NBB and LBF leagues.

	**NBB**		**LBF**	
	**Men**	**Women**	**Men**	**Women**
Black coaches	3	0	2	3
White coaches	66	0	30	5
Total	69	0	32	8
General total	109			

NBB, Novo Basquete Brasil (New Basketball Brazil); LBF, Liga de Basquete Feminino (Womens' Basketball League).

The authors apologize for this error and state that this does not change the scientific conclusions of the article in any way. The original article has been updated.

