# Supplementary material for: Of the few Black coaches in Brazilian professional basketball leagues: approaches to racism
Source: Front Psychol. 2025 Apr 10;16:1511967. doi: 10.3389/fpsyg.2025.1511967 (PMC12018311; doi:10.3389/fpsyg.2025.1511967)
Supplement: Supplementary file 1 [file Data_Sheet_1.docx]

**Interview guide**

Contextualization: We aim to understand your experience as a Black coach. We would like to know how you got to where you are today, the people and events that influenced you, your past and current experiences, and your journey to your current position.

1. How do you identify yourself? Do you consider yourself Black?
2. Where were you born? Did you grow up in the same place? How did you get involved in sports?
3. How long have you been a coach?
4. Could you describe your journey as a coach? How did your career begin, evolve, and lead you to where you are today?
5. Compared to the career paths of your white colleagues, do you think your journey was different? In what ways?
6. Regarding the opportunities you had to achieve your career goals, how would you evaluate them?
7. Among your colleagues in the coaching profession, do you see other Black people?
8. Do you think this situation needs to change? How could it change? What do you believe needs to be done?
9. Have you participated in or heard of any initiatives aimed at promoting change in this regard?
10. We have talked about the past and present so far. Now, thinking about the future, how do you evaluate your prospects and opportunities to achieve new goals?
11. What advice would you give to Black individuals who want to start or are starting their journey as coaches? What have you learned in your career that could help guide beginners?
12. Does being a Black woman make a difference in your career?
13. Have we missed anything? Is there anything else you would like to share that we did not ask about?
