# Supplementary figures and images for: Of the few Black coaches in Brazilian professional basketball leagues: approaches to racism
Source: Front Psychol. 2025 Apr 10;16:1511967. doi: 10.3389/fpsyg.2025.1511967 (PMC12018311; doi:10.3389/fpsyg.2025.1511967)

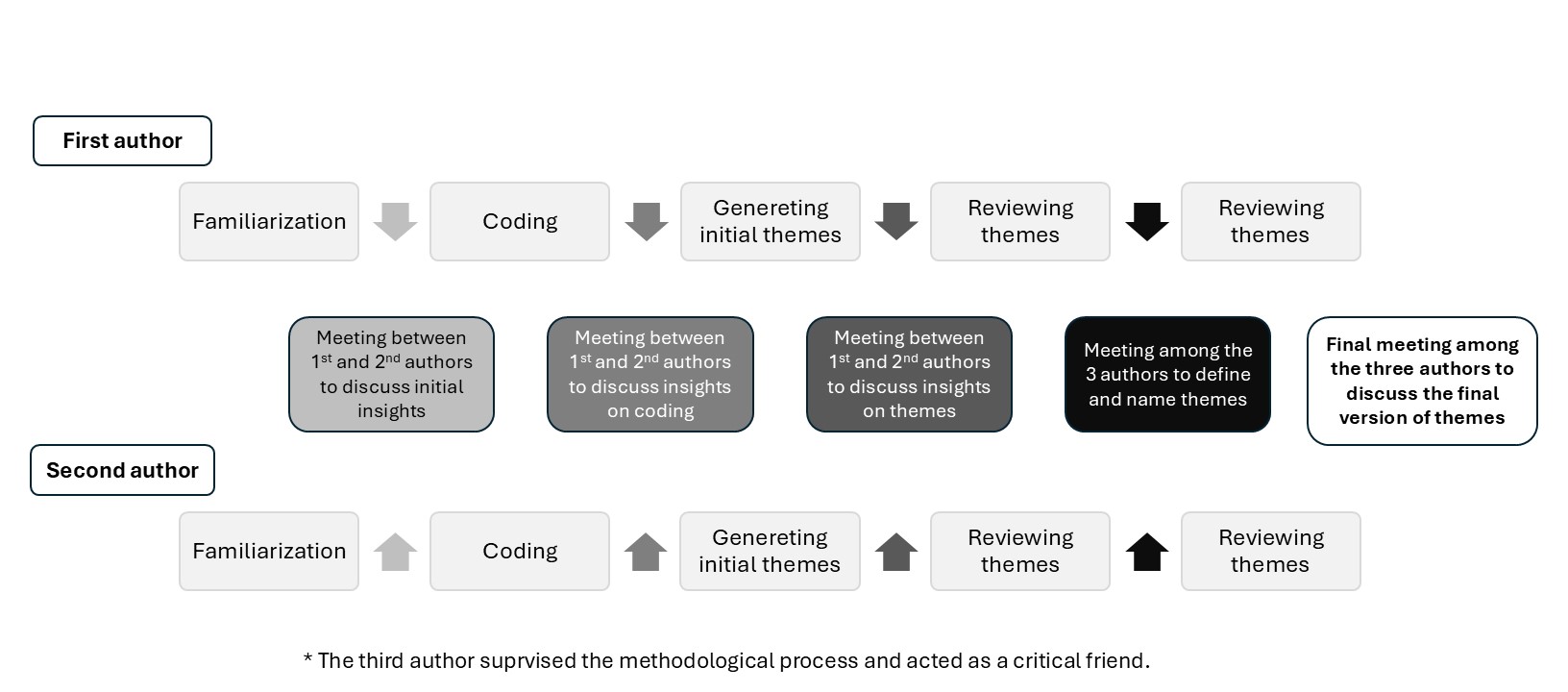

Supplement: Supplementary file 2 [file Image_1.jpg]
